# Zoledronate blocks geranylgeranylation not farnesylation to suppress human osteosarcoma U2OS cells metastasis by EMT via Rho A activation and FAK-inhibited JNK and p38 pathways

**DOI:** 10.18632/oncotarget.7138

**Published:** 2016-02-02

**Authors:** Hsin-Lin Cheng, Chiao-Wen Lin, Jia-Sin Yang, Ming-Ju Hsieh, Shun-Fa Yang, Ko-Hsiu Lu

**Affiliations:** ^1^ Institute of Medicine, Chung Shan Medical University, Taichung 40201, Taiwan; ^2^ Department of Medical Research, Chung Shan Medical University Hospital, Taichung 40201, Taiwan; ^3^ Institute of Oral Sciences, Chung Shan Medical University, Taichung 40201, Taiwan; ^4^ Department of Dentistry, Chung Shan Medical University Hospital, Taichung 40201, Taiwan; ^5^ Cancer Research Center, Changhua Christian Hospital, Changhua 500, Taiwan; ^6^ Department of Orthopedics, Chung Shan Medical University Hospital, Taichung 40201, Taiwan; ^7^ School of Medicine, Chung Shan Medical University, Taichung 40201, Taiwan

**Keywords:** geranylgeranylation, metastasis, Rho A, U2OS, zoledronate

## Abstract

Zoledronate is a standard treatment for preventing skeletal complications of osteoporosis and some types of cancer associated with bone metastases, but we little know whether the effect of zoledronate on metastasis of osteosarcoma. Here, we investigated the inhibitory effects of zoledronate on cell viability, motility, migration and invasion of 4 osteosarcoma cell lines (Saos2, MG-63, HOS and U2OS) by affecting cell morphology, epithelial-mesenchymal transition (EMT) and cytoskeletal organization as well as induction of E-cadherin and reduction of N-cadherin with activation of transcription factors Slug and Twist, especially in U2OS cells. Zoledronate decreased JNK and p38 phosphorylation and upper streams of focal adhesion kinase (FAK) and Src to suppress the motility, invasiveness and migration of U2OS cells. In addition to zoledronate-inhibited Rho A and Cdc42 membrane translocation and GTPγS activities, the anti-metastatic effects in U2OS cells including inhibition of adhesion were reversed by geranylgeraniol, but not farnesol. In conclusion, Zoledronate blocks geranylgeranylation not farnesylation to suppress human osteosarcoma U2OS cell-matrix and cell-cell interactions, migration potential, the invasive activity, and the adhesive ability by EMT via Rho A activation and FAK-inhibited JNK and p38 pathways.

## INTRODUCTION

Osteosarcoma, the most common histological form of primary bone cancer, consists of approximately 20% of all primary bone cancers [1, 2]. According to the new chemotherapy protocols, surgical techniques, and radiological staging, the combination of surgery and chemotherapy has enabled promoted frequency of the long-term survival to an approximate 68% in 2009 and limbsparing surgery in the treatment of osteosarcoma [2, 3]. However, the metastatic ability of osteosarcoma is accountable for poor prognoses and high mortality rates.

Metastasis, including several events collectively termed the invasion-metastasis cascade [4], results in the detachment of tumor cells, motility, degradation of the extracellular matrix (ECM), invasion, migration, adhesion to endothelial cells, and the reestablishment of growth at a distant site [5]. Initially, the epithelial-mesenchymal transition (EMT) combines loss of epithelial cell junction proteins (e.g. E-cadherin) and the gain of mesenchymal markers, such as N-cadherin and Vimentin [6], as well as activation of transcription factors including Slug, Snail and Twist [7]. Besides, matrix metalloproteinase (MMP)-2 (gelatinase A, 72 kDa) and MMP-9 (gelatinase B, 92 kDa) plays importantly in the process of tumor cell migration and invasion [8].

Invasion of individual tumor cells can proceed via the integrin-dependent (mesenchymal invasion) or integrin-independent and Ras homolog gene family (Rho)/Rho-associated protein kinase-dependent (amoeboid invasion) mechanism [9]. Integrins, consisting of α- and β-subunits and their receptors, are a family of transmembrane glycoprotein adhesion receptors that activate cell-matrix and cell-cell adhesion, the first step of metastasis [10]. Small Rho family GTPases control multiple cellular functions such as adhesion, spreading, migration, and division and are involved in all stages during cancer progression [11]. Rho GTPase-dependent regulation of cellular motility and migration is associated with the control of dynamic reorganization of the actin cytoskeleton and the mediation of the formation of specific actin structures [12]. Rho, member A (Rho A), Rac-1, and Cdc42 are prototypical members of the Rho family representing three canonical subgroups [11, 13]. Specifically, Cdc42 and Rac-1 revealed redundant effects toward cell polarization and lamellipodium formation, characteristic processes of mesenchymal motility [14]. Rho A, Cdc42, and Rac-1 shuttle between their inactive GDP– and active GTP–bound forms to regulate the dynamics of the actin cytoskeleton, cell motility, cadherin-dependent adhesion, and cell proliferation [15, 16].

Malignant cells often decrease the levels of E-cadherin, which is anchored to the cytoskeleton. In particular, changes in the organization of the actin cytoskeleton, which is implicated in adhesion-induced, integrin-activated focal adhesion kinase (FAK), lead to remarkable changes in the tyrosine phosphorylation of several signaling proteins localized at the focal adhesion complex [17]. FAK and the nonreceptor protein tyrosine kinase Src connections forming a dual kinase complex are important for the control of cell motility and invasion [18]. In addition to regulating cell adhesion to the ECM and proteolytic enzyme activities to basement membrane degradation, integrins activate kinases to phosphorylate cytoskeletal proteins and to manage stress-fiber formation, cellular shape and migration [19]. E-cadherin–based adherens junctions interact with catenins to modulate cell-cell adhesion [20].

Zoledronate or zoledronic acid, a nitrogen-containing bisphosphonate, is the most potent member in the bisphosphonate family as a standard treatment for preventing skeletal complications of osteoporosis and some types of cancer associated with bone metastases. Apart from anti-metastatic potential, zoledronate has direct, indirect or immune anti-tumor effects and blocks tumor cell adhesion to make tumor cells more susceptible to the cytotoxic effects of chemotherapy [21, 22]. The mechanism comprises impairing migration and invasion, modulating immune response and anti-angiogenesis effect, inhibiting tumor proliferation, and inducing cell apoptosis [23]. Generally, zoledronate inhibits the formation of farnesyl diphosphate (FPP) and geranylgeranyl diphosphate (GGPP) by suppressing FPP synthase and GGPP synthase [24]. This suppression results in the inhibition of prenylation of small GTPases such as Ras, Rho, and Rac because FFP and GGPP are required for the posttranslational prenylation of small signaling GTPase in transducing extracellular signal for cell function such as cell proliferation, migration, and invasion [25, 26]. Importantly, preclinical studies reported that zoledronate may prevent lung metastases of osteosarcoma *in vivo* [27-29], however, the anti-metastatic effect of zoledronate in human osteosarcoma is few to be investigated. Here, we proposed that zoledronate may suppress osteosarcoma cells to exert anti-metastatic effects, and further explored the underlying mechanisms involved.

## RESULTS

### Cytotoxicity of zoledronate in 4 osteosarcoma cells

After 24 h treatment, 4 osteosarcoma (Saos2, MG-63, HOS and U2OS) cells viability in the presence of concentrations of 25, 50, 75 and 100 μM zoledronate was not significantly different to that of controls (0 μM) in the microculture tetrazolium assay (see Supplementary Material online, Figure S1A). Thus, a 24 h treatment with zoledronate up to 100 μM had no cytotoxic effect on 4 osteosarcoma cells. We used this concentration range for zoledronate in all subsequent experiments to investigate its anti-metastatic properties.

### Zoledronate inhibits 4 osteosarcoma cells motility, invasiveness and migration

**Figure 1:**
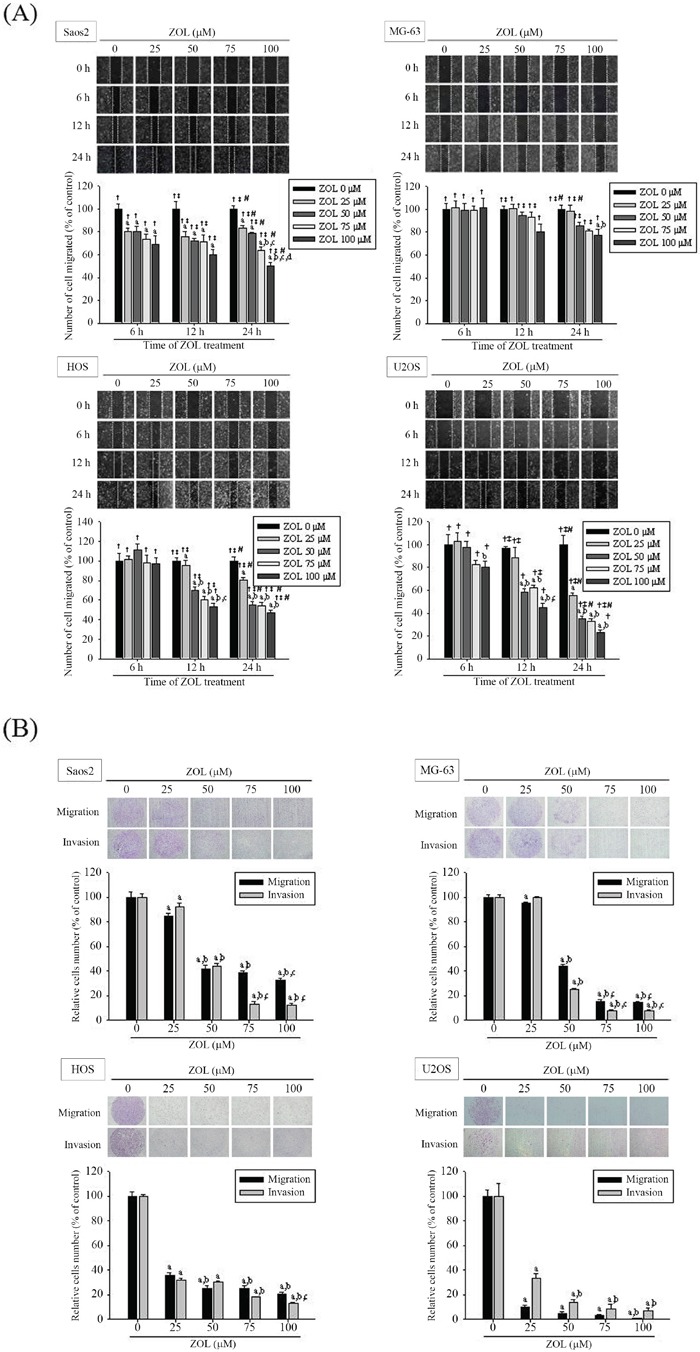
Effects of zoledronate on the wound healing, cell migration and invasion assays in 4 osteosarcoma (Saos2, MG-63, HOS and U2OS) cells **A.** The wound healing assay after different concentrations (0, 25, 50, 75, and 100 μM) and different time (0, 6, 12, 24 h) of zoledronate treatment and **B.** the cell migration and invasion assays after different concentrations (0, 25, 50, 75, and 100 μM) of zoledronate treatment for 24 h in 4 osteosarcoma cells were measured as described in the Materials and Methods section. Concentration effects: wounding healing (Saos2: *F* = 144.888, *p* < 0.001. MG-63: *F* = 6.9, *p* = 0.006. HOS: *F* = 153.379, *p* < 0.001. U2OS: *F* = 160.048; *p* < 0.001); cell migration (Saos2: *F* = 321.366, *p* < 0.001. MG-63: *F* = 3139.028, *p* < 0.001. HOS: *F* = 630.053, *p* < 0.001. U2OS: *F* = 873.706, *p* < 0.001); invasion (Saos2: *F* = 1005.528, *p* < 0.001. MG-63: *F* = 5081.399, *p* < 0.001. HOS: *F* = 3031.602, *p* < 0.001. U2OS: *F* = 165.519, *p* < 0.001). ^a^Significantly different, *p* < 0.05, when compared with the vehicle group. ^b^Significantly different, *p* < 0.05, when compared with 25 μM. ^c^Significantly different, *p* < 0.05, when compared with 50 μM. ^d^Significantly different, *p* < 0.05, when compared with 75 μM. Time effects: wounding healing (Saos2: *F* = 239.005, *p* < 0.001. MG-63 *F* = 58.474, *p* < 0.001. HOS: *F* = 273.078, *p* < 0.001. U2OS: *F* = 114.156, *p* < 0.001.) †Significantly different, *p* < 0.05, when compared with 0 h. ‡Significantly different, *p* < 0.05, when compared with 6h. #Significantly different, *p* < 0.05, when compared with 12h.

In the wound-healing assay, zoledronate significantly attenuated cell motility of 4 osteosarcoma cells both dose- and time-dependently (Figure 1A). Also, both modified Boyden chamber with or without Matrigel assays showed that zoledronate significantly inhibited the migration activity and invasive potential in 4 osteosarcoma cells dose-dependently (Figure 1B). Taken together, zoledronate seemed to be the most potent in U2OS cells.

### Zoledronate has no effect on MMP-2 and MMP-9 of 4 osteosarcoma cells

In gelatin zymography, different concentrations of 25, 50, 75 and 100 μM of zoledronate did not show any different effect to that of control on MMP-2 and MMP-9 levels in 4 osteosarcoma cells (Supplementary Material online, Figure S1B). Similarly, no significant effects at different concentrations of 0, 25, 50, 75 and 100 μM of zoledronate on MMP-2 and MMP-9 expressions were noted in western blotting analysis (Supplementary Material online, Figure S1C).

### Zoledronate affects 4 osteosarcoma cells morphology and EMT

**Figure 2:**
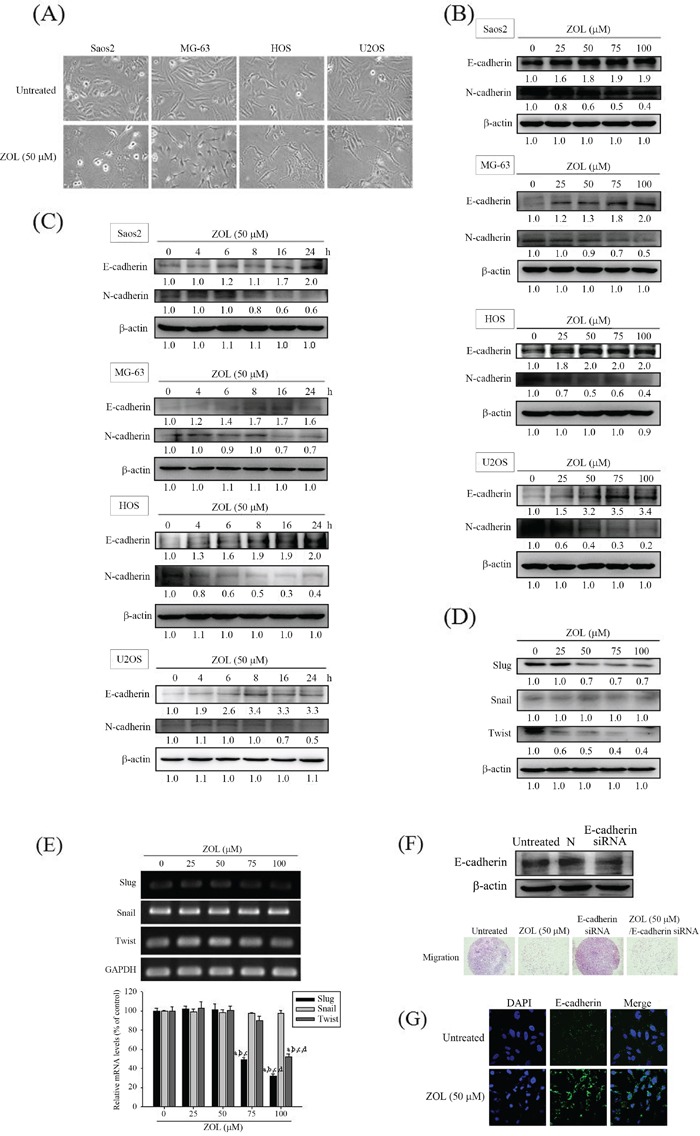
Effects of zoledronate on cell morphology and the EMT in 4 osteosarcoma (Saos2, MG-63, HOS and U2OS) cells **A.** Cell morphology changes after 50 μM zoledronate treatment for 24 h in 4 osteosarcoma cells were observed. **B.** Expressions of E-cadherin and N-cadherin after different concentrations (0, 25, 50, 75, and 100 μM) and **C.** different time (0, 4, 6, 8, 16, 24 h) of zoledronate treatment in 4 osteosarcoma cells were measured by western blot analysis. **D.** Expressions and **E.** m-RNA levels of transcriptional factors Slug, Snail and Twist after different concentrations (0, 25, 50, 75, and 100 μM) of zoledronate treatment for 24 h in U2OS cells were measured by western blot analysis and RT-PCR, respectively. Concentration effects (Slug: *F* = 282.201. *p* < 0.001. Snail: *F* = 0.543, *p* = 0.708. Twist: *F* = 58.766. *p* < 0.001). ^a^Significantly different, *p* < 0.05, when compared with the vehicle group. ^b^Significantly different, *p* < 0.05, when compared with 25 μM. ^c^Significantly different, *p* < 0.05, when compared with 50 μM. ^d^Significantly different, *p* < 0.05, when compared with 75 μM. **F.** The cell migration assays after 50 μM zoledronate, siRNA E-cadherin or both treatments for 24 h in U2OS cells were measured. **G.** Cytoskeleton E-cadherin arrangement after 50 μM zoledronate treatment for 24 h in U2OS cells was analyzed by immunofluorescence staining.

As shown in Figure 2A, 4 osteosarcoma cells became shrunken after 50 μM zoledronate treatment. Using western blot analysis, we found that zoledronate increased the E-cadherin expression but attenuated the N-cadherin expression in 4 osteosarcoma cells both in dose- and time-dependent appearance (Figure 2B & 2C). Again, zoledronate seemed to possess the most potency of activating E-cadherin and suppressing N-cadherin expressions in U2OS cells among 4 osteosarcoma cell lines. For examining the underlying mechanisms, therefore, we chose 50 μM zoledronate in all subsequent experiments.

To explore whether zoledronate interferes with EMT transcription factors (Slug, Snail and Twist) in U2OS cells, western blotting analysis and RT-PCR were used. We observed that zoledronate decreased protein expressions and mRNA levels of Slug and Twist dose-dependently, respectively, but did not affect the protein expression and the m-RNA level of Snail (Figure 2D & 2E). E-cadherin is regarded as a gatekeeper of the epithelial state in various epithelial cell types, so we next used siRNA directly against the E-cadherin expression and found that it promoted U2OS cell migration but the effect was attenuated by zoledronate (Figure 2F). Using immunofluorescence staining, the induction effect of E-cadherin-induced rearrangement of cytoskeletal organization by 50 μM zoledronate in U2OS cells was also noted (Figure 2G).

### Zoledronate decreases c-Jun N-terminal kinase (JNK) and p38 phosphorylation of U2OS cells

**Figure 3:**
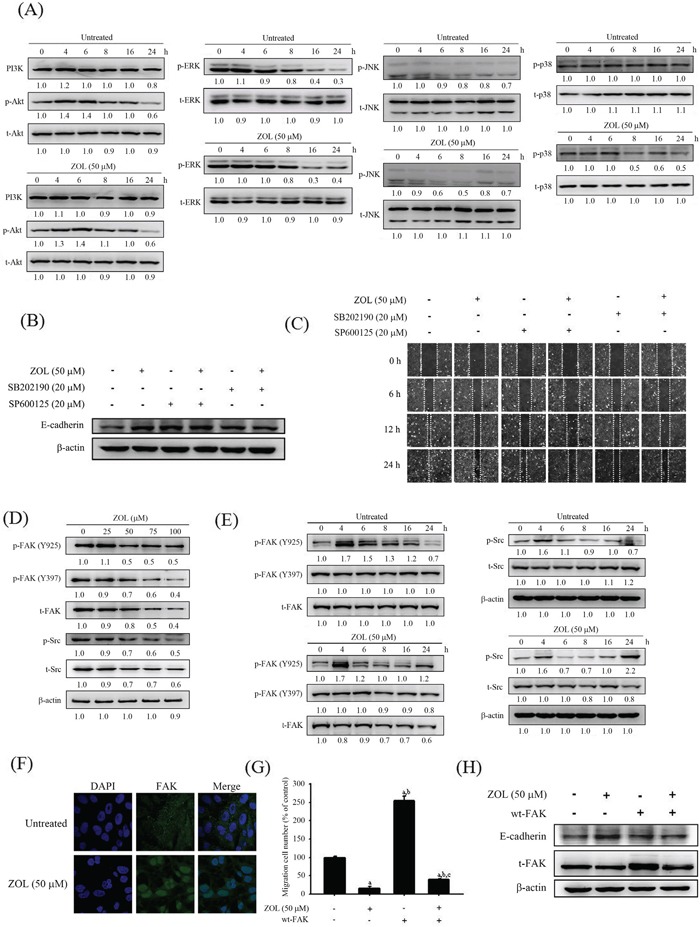
Effects of 50 μM zoledronate on the expressions of FAK, Src, MAPKs and PI3K-Akt in U2OS cells **A.** Expressions of ERK, JNK, p38, PI3K and Akt and their phosphorylation after 50 μM zoledronate treatment for different time (0, 4, 6, 8, 16 and 24 h) in U2OS cells were measured by western blot analysis. **B.** The E-cadherin expression in western blotting and **C.** the wound healing assay after 50 μM zoledronate, the JNK inhibitor (SP600125), the p38 inhibitor (SB202190), or 50 μM zoledronate plus one inhibitor treatments for 24 h in U2OS cells were measured. **D.** Expressions of FAK, Src and their phosphorylation after different concentrations (0, 25, 50, 75, and 100 μM) treatment for 24 h and **E.** after 50 μM zoledronate treatment for different time (0, 4, 6, 8, 16 and 24 h) in U2OS cells were measured by western blot analysis. **F.** FAK changes after 50 μM zoledronate treatment in U2OS cells were analyzed by immunofluorescence staining. **G.** The cell migration assay and **H.** the E-cadherin expression after 50 μM zoledronate, wt-FAK or both treatments in U2OS cells were measured. *F* = 793.922, *p* < 0.001. ^a^Significantly different, *p* < 0.05, when compared with the vehicle group. ^b^Significantly different, *p* < 0.05, when compared with the zoledronate-treated group. ^c^Significantly different, *p* < 0.05, when compared with the wt-FAK-treated group.

In order to investigate the molecular mechanisms further, extracellular signal-regulated protein kinases and phosphatidylinositol 3-kinase (PI3K)-Akt pathways were detected in each group using western blot analysis. In the presence of 50 μM zoledronate for 6-8 hours, phosphorylation of JNK and p38 was markedly reduced in U2OS cells, while there was no obvious influence of zoledronate on extracellular signal-regulated protein kinase (ERK) and PI3K-Akt phosphorylation (Figure 3A). Neither total ERK nor PI3K-Akt was changed. These results suggested involvement of JNK and p38 signal pathways in the effects of zoledronate on U2OS cells. Using inhibitors of JNK (SP600125) and p38 (SB202190), the induction of the E-cadherine expression and inhibition of cell motility in the wound healing assay by 50 μM zoledronate in U2OS cells was further believed through down-regulation of JNK and p38 phosphorylation (Figure 3B & 3C).

### Zoledronate decreases t-FAK, p-FAK Tyr397, p-FAK Tyr925 and Src expressions of U2OS cells

For better defining the role of zoledronate on upper stream FAK family, western blot analysis was used and showed that zoledronate significantly reduced t-FAK, p-FAK Tyr397, p-FAK Tyr925 and Src expressions in dose- and time-dependent appearance (Figure 3D & 3E). Using immunofluorescence staining, we also observed the effect of FAK reduction by 50 μM zoledronate in U2OS cells (Figure 3F). Next, wild-type FAK (wt-FAK) was used and showed that wt-FAK promoted U2OS cell migration and reduced the E-cadherin expression and these effects were attenuated by 50 μM zoledronate. (Figure 3G & 3H).

### Geranylgeraniol (GGOH) reverses the anti-metastatic effects of zoledronate in U2OS cells

**Figure 4:**
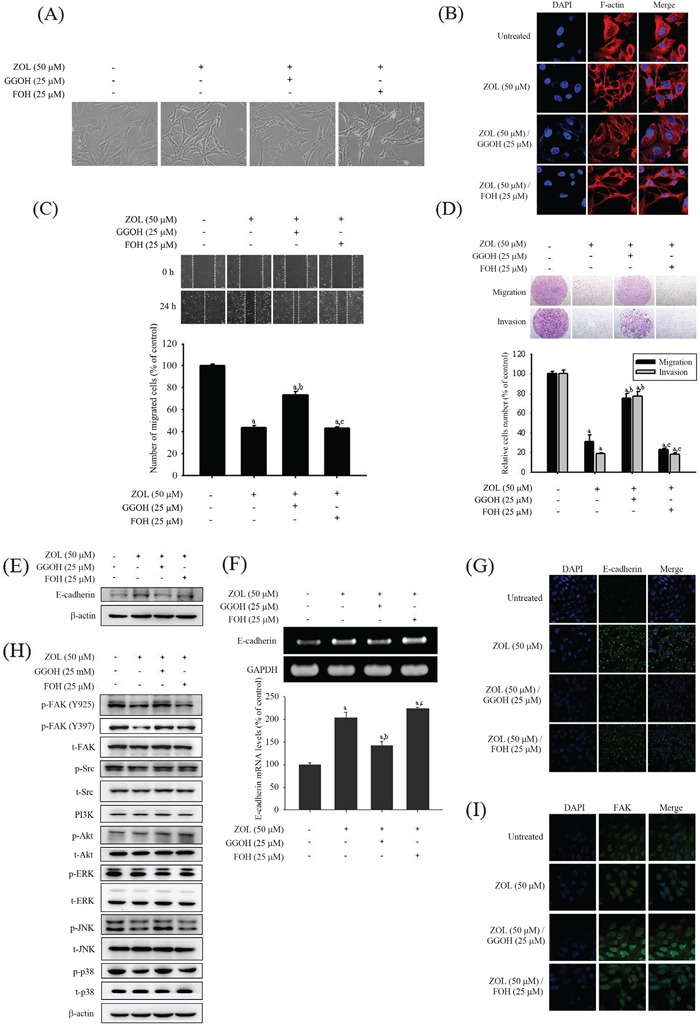
Effects of GGOH and FOH on zoledronate-induced anti-metastatic effects in U2OS cells **A.** Cell morphology, **B.** cytoskeleton F-actin arrangement, **C.** the wound healing assay, **D.** the cell migration and invasion assays, **E.** the protein expression and **F.** the m-RNA level of E-cadherin, **G.** E-cadherin changes in immunofluorescence staining, **H.** expressions of MAPKs, PI3K, Akt, FAK, Src and their phosphorylation, and **I.** FAK changes in immunofluorescence staining after 50 μM zoledronate, 50 μM zoledronate plus 25 μM GGOH, or 50 μM zoledronate plus 25 μM FOH treatments for 24 h were observed in U2OS cells. Wound healing: *F* = 604.167, *p* < 0.001; migration: *F* = 224.704, *p* < 0.001; invasion: *F* = 499.471, *p* < 0.001; E-cadherin: *F* = 130.029, *p* < 0.001. ^a^Significantly different, *p* < 0.05, when compared with the vehicle group. ^b^Significantly different, *p* < 0.05, when compared with the zoledronate-treated group. ^c^Significantly different, *p* < 0.05, when compared with the zoledronate plus GGOH-treated group.

As demonstrated in Figure 4A, the disturbance of cell morphology in U2OS cells induced by 50 μM zoledronate and the cells developed a pronounced spindle-like morphological appearance. The change was marked restored in the presence of exogenous 25 μM GGOH, whereas there was no obvious influence in the presence of exogenous 25 μM farnesol (FOH). To further investigate the cytoskeletal organization of U2OS cells, F-actin cytoskeletons were assessed by staining with Texas Red-X phalloidin. After counterstaining with DAPI for nuclei, U2OS cells became shrunken and F-actin fibers apparently condensed upon 50 μM zoledronate treatment, comparing with the control plate (Figure 4B). The inhibitory effects of 50 μM zoledronate against motility, invasiveness and migration in U2OS cells were also reversed by addition of 25 μM GGOH, but not by addition of 25 μM FOH (Figure 4C & 4D). These results suggested that zoledronate attenuated cell-matrix and cell-cell interactions, migration potential, and invasive activity of U2OS cells by blocking of geranylgeranylation, but not farnesylation.

Furthermore, the effects of induction of E-cadherin protein expression and mRNA level in western blot analysis and RT-PCR, respectively, and that in immunofluorescence staining by 50 μM zoledronate were all reversed by addition of 25 μM GGOH, but not by addition of 25 μM FOH (Figure 4E, 4F & 4G). Similarly, both western blot analysis and immunofluorescence staining showed that exogenous GGOH could reverse the inhibitory effects of FAK and Src expressions as well as JNK and p38 phosphorylation by 50 μM zoledronate in U2OS cells but FOH could not. This indicated that zoledronate blocked geranylgeranylation not farnesylation to suppress FAK and Src expressions and JNK and p38 phosphorylation in U2OS cells (Figure 4H & 4I).

### GGOH reverses zoledronate-inhibited membrane translocation and GTPγS of Rho A and Cdc42 of U2OS cells

**Figure 5:**
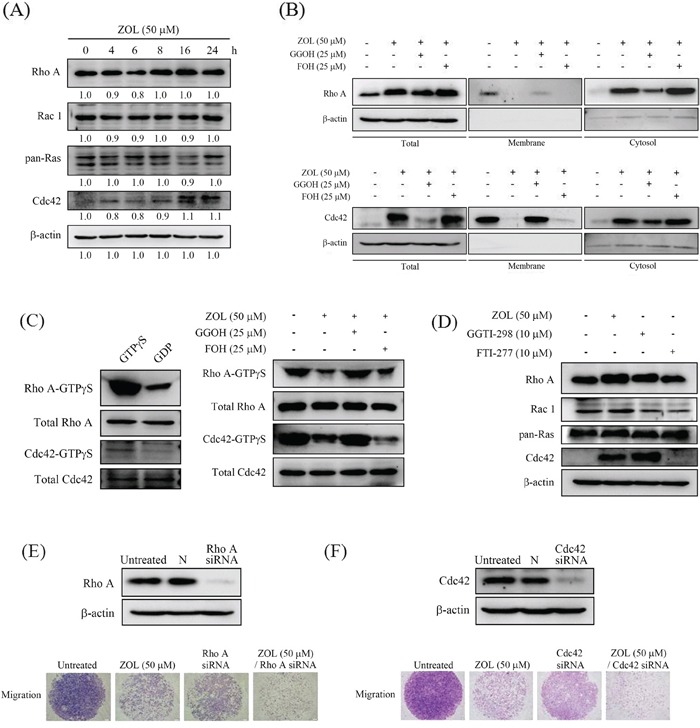
Effects of GGOH and FOH on zoledronate-suppressed membrane translocation and GTPγS activities of Rho A and Cdc42 in U2OS cells **A.** Expressions of Rho A, Rac 1, pan-Ras and Cdc42 after 50 μM zoledronate treatment for different time (0, 4, 6, 8, 16 and 24 h) in U2OS cells were measured by western blot analysis. **B.** Expressions of Rho A and Cdc42 after 50 μM zoledronate, 50 μM zoledronate plus 25 μM GGOH, or 50 μM zoledronate plus 25 μM FOH treatments for 24 h were measured in cell lysate, membrane and cytosol of U2OS cells by western blot analysis. **C.** Activities of Rho A-GTPγS and Cdc42-GTPγS after 50 μM zoledronate, 50 μM zoledronate plus 25 μM GGOH, or 50 μM zoledronate plus 25 μM FOH treatments for 24 h were measured in U2OS cells as described in the Materials and Methods section. **D.** Expressions of Rho A, Rac 1, pan-Ras and Cdc42 after 50 μM zoledronate, 10 μM GGOH inhibitor (GGTI-298), or 10 μM FOH inhibitor (FTI-277) treatments for 24 h in U2OS cells were measured by western blot analysis. **E.** The cell migration assays after 50 μM zoledronate, siRNA Rho A or both treatments, and **F.** after 50 μM zoledronate, siRNA Cdc42 or both treatments in U2OS cells were measured.

Similar to induction of E-cadherin, 50 μM zoledronate seemed to elevate Rho A and Cdc42 expressions in U2OS cells after 24 h treatment, but no effect on Rac-1 and pan-Ras expressions (Figure 5A). Actually, we could found that expressions of Rho A and Cdc42 declined at 6 h treatment of 50 μM zoledronate in U2OS cells. After 24 h treatment of 50 μM zoledronate in U2OS cells, we further extracted cell membrane proteins and analyzed Rho A and Cdc42 expressions in the membrane and in the cytosol, respectively. Interestingly, Rho A and Cdc42 expressions significantly showed reduction in the cell membrane with the concomitant increase in the cytosol, strongly implying Rho A and Cdc42 translocation from the cell membrane to the cytosol in response to zoledronate (50 μM, 24 h) (Figure 5B). Also, this zoledronate-inhibited Rho A and Cdc42 membrane translocation was reversed by addition of 25 μM GGOH, but not by addition of 25 μM FOH, implicating the effect specific to geranylgeranylation inhibition.

Moreover, 50 μM zoledronate markedly decreased Rho A-GTPγS and Cdc42-GTPγS activities in the cell membrane of U2OS cells and the inhibitory effect was consistent with translocation from the cell membrane to the cytosol. The effect was also restored by addition of 25 μM GGOH, but not by addition of 25 μM FOH, still implicating the effect specific to geranylgeranylation inhibition (Figure 5C). Using geranylgeranyltransferase I (GGTase I) inhibitor GGTI-298 and farnesyltransferase (FTase) inhibitor FTI-277 to block geranylgeranylation and farnesylation, respectively, we interestingly observed that 10 μM GGTI-298 increased Rho A and Cdc 42 expressions in U2OS cells, but not on Rac-1 and pan-Ras, mimicking the effect of 50 μM zoledronate, whereas 10 μM FTI-277 did not influence Rho A and Cdc 42 expressions. This further indicated that the effect of zoledronate in U2OS cells was through blocking geranylgeranylation not farnesylation (Figure 5D). Additionally, siRNA knockdown of either Rho A or Cdc42 diminished U2OS cell migration and exaggerated the inhibitory effect of cell migration by 50 μM zoledronate, further suggesting that zoledronate decreased Rho A and Cdc42 activation to suppress U2OS cell migration (Figure 5E & 5F).

### GGOH reverses zoledronate-inhibited α_4_-,α_6_- and β_1_-integrins and adhesion of U2OS cells

**Figure 6:**
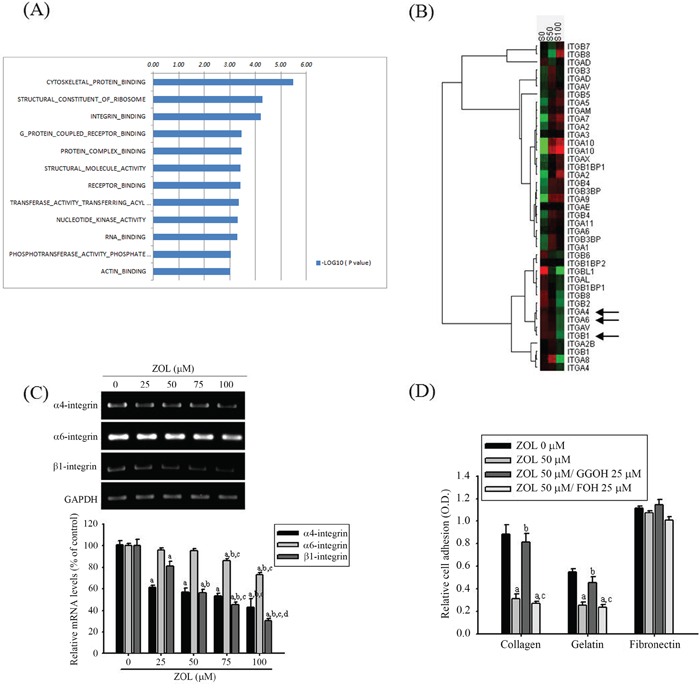
Effects of GGOH and FOH on zoledronate-suppressed α_4_-, α_6_- and β_1_-integrins and adhesion in U2OS cells **A.** The geneset list of gene ontology enrichment analysis shows the enriched bar chart of cellular function categories in U2OS cells treated with 100 μM zoledronate for 6 h. Log10 ratio indicated treated U2OS cells as compared with untreated cells. **B.** After treating with 50 (S50) and 100 μM (S100) zoledronate in U2OS cells for 24 h, analyses of the integrin family gene expression were pooled and used to generate the heat map that shows the result of the two-way hierarchical clustering of genes and samples. Each row represents a gene and each column represents a sample. The gene clustering tree is shown on the left, and the sample clustering tree appears at the top. Increased and decreased genes are represented in red and green, respectively. ITGA4 (α_4_-integrin), ITGA6 (α_6_-integrin) and ITGB1 (β_1_-integrin) genes were significantly inhibited (arrow). **C.** U2OS cells were treated with zoledronate (0, 25, 50, 75 and 100 μM) for 24 h before being subjected to RT-PCR for mRNA expressions of α_4_-, α_6_- and β_1_-integrins. α_4_-integrin: *F* = 70.869, *p* < 0.001; α_6_-integrin: *F* = 98.511, *p* = 0.006; β_1_-integrin: *F* = 181.995, *p* < 0.001. ^a^Significantly different, *p* < 0.05, when compared with the vehicle group. ^b^Significantly different, *p* < 0.05, when compared with 25 μM. ^c^Significantly different, *p* < 0.05, when compared with 50 μM. ^d^Significantly different, *p* < 0.05, when compared with 75 μM. **D.** The cell adhesion assays after 50 μM zoledronate, 50 μM zoledronate plus 25 μM GGOH, or 50 μM zoledronate plus 25 μM FOH treatments for 24 h were observed in U2OS cells. Collagen: *F* = 74.826, *p* < 0.001; gelatin: *F* = 36.808, *p* < 0.001; fibronectin: *F* = 3.682, *p* = 0.062. ^a^Significantly different, *p* < 0.05, when compared with the vehicle group. ^b^Significantly different, *p* < 0.05, when compared with the zoledronate-treated group. ^c^Significantly different, *p* < 0.05, when compared with the zoledronate plus GGOH-treated group.

Using microarray analysis, the inhibitory effects of zoledronate on clustering of gene profiles in U2OS cells is shown in Figure 6A. Since integrins function as the major cell receptor for extracellular matrix protein and integrin binding genes showed significant inhibition by 100 μM zoledronate, we subsequently analyzed the integrin family gene expressions. Hierarchical clustering was carried out to illustrate the distinguishable gene expression profiles between the 3 groups without, with 50 or 100 μM zoledronate treatments (Figure 6B). In hierarchical clustering of gene profiles, zoledronate seemed to have the inhibitory influence on expressions of α_4_-, α_6_- and β_1_-integrins in U2OS cells. To validate the results of our microarray analysis, RT-PCR was performed to confirm the changes. U2OS cells were pre-treated with increasing concentrations (0, 25, 50, 75 and 100 μM) of zoledronate for 24 h and the inhibitory effects of α_4_-, α_6_- and β_1_-integrins were all in a dose-dependent manner (Figure 6C). To further assess whether zoledronate decreases cell adhesion to extracellular matrix components, U2OS cells were pre-treated with or without 50 μM of zoledronate prior to the adhesion assay on collagen type 1 (α_2_β_1_-ligand), gelatin (heat-denatured collagen, α_5_β_3_-ligand), or fibronectin (α_5_β_1_-ligand). Consequently, zoledronate significantly impaired cell adhesion on collagen type 1 and gelatin, but not on fibronectin in U2OS cells (Figure 6D). Consistent with our previous findings, the zoledronate-inhibited ant-adhesive abilities on collagen type I and gelatin were reversed by addition of 25 μM GGOH, but not by addition of 25 μM FOH.

## DISCUSSION

In the present study, zoledronate at concentrations without cytotoxicity (up to 100 μM) inhibited tumor cell motility, invasion and migration in 4 osteosarcoma cell lines (Saos2, MG-63, HOS and U2OS). Generally, tumor cell invasion requires both cell migration and digestion of the basement membrane by MMPs [6-8, 30], but zoledronate could not affect expressions and m-RNA levels of MMP-2 and -9 in 4 osteosarcoma cell lines. Zoledronate inhibited cell-matrix and cell-cell interactions, migration potential, and the invasive activity by induction of E-cadherin and reduction of N-cadherin with activation of transcription factors Slug and Twist in 4 osteosarcoma cells, especially in U2OS cells and seemed to possess the most potency in U2OS cells. Therefore, we chose 50 μM zoledronate in all subsequent experiments to examine the underlying mechanisms. We silenced the E-cadherin protein using siRNA and observed that it promoted U2OS cell migration and the effect was attenuated by zoledronate, further indicating zoledronate-induced E-cadherin to suppress U2OS cell migration.

**Figure 7:**
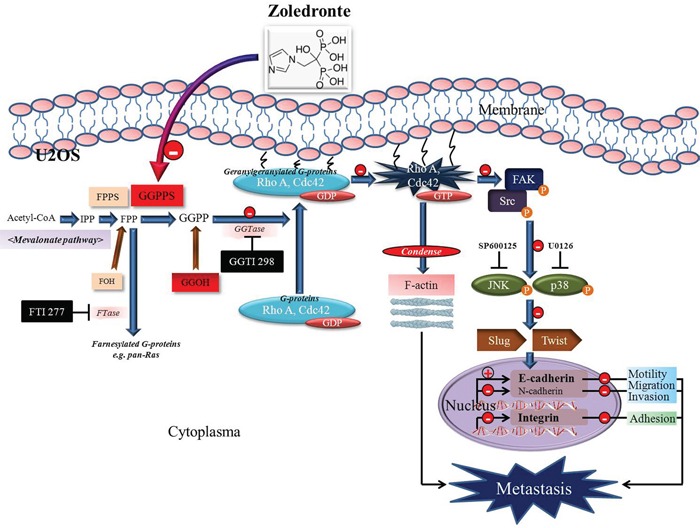
The schematic representation of anti-metastasis effects of zoledronate in human osteosarcoma U2OS cells Zoledronate blocks geranylgeranylation not farnesylation to suppress human osteosarcoma U2OS cell-matrix and cell-cell interactions, migration potential, the invasive activity, and the adhesive ability by EMT via Rho A activation and FAK-inhibited JNK and p38 pathways.

Src is capable of modulating cell migration and invasion through interaction with integrins, the FAK, and regulators of the family of Rho-GTPases [31]. Although zoledronate inhibits prostate cancer proliferation through ERK/Akt inactivation and synergizes with panobinostat in prostate cancer and multiple myeloma models by increasing ROS and modulating mevalonate and p38-MAPK pathways, zoledronate inhibits the MAPK and induced apoptosis of hepatoma cells [11, 32, 33]. However, we demonstrated that the anti-metastatic effect of zoledronate in U2OS cells was through Rho A activation and FAK-inhibited JNK and p38 pathways, but neither total ERK nor PI3K-Akt influence. Indeed, zoledronate interfered with cell morphology, EMT and cytoskeletal organization in U2OS cells and blocked the enzyme of geranylgeranylation not farnesylation of the mevalonate pathway to suppress U2OS cell migration. We further investigated that zoledronate caused accumulation of Rho A and Cdc42 in the cytosol relative to the membrane and also increased the total amount of cellular Rho A, compatible with other reports describing up-regulation of Rho-families GTPases following depletion of mevalonate-derived isoprenoids [26, 34]. The addition of GGOH but not FOH, prevented zoledronate-activated Rho A and Cdc42 in total cell and the cytosol and zoledronate-suppressed Rho A- and Cdc42-GTPγS activities in the cell membrane. The siRNA silencing of Rho A and Cdc42 declined U2OS cell migration and the effect was amplified by zoledronate, further implying the decreasing Rho A and Cdc42 activation in suppression of U2OS cell migration (Figure 7).

Cancer cell adhesion is an important step of the metastatic cascade that is essential for the cancer to establish persistence at the site of metastasis, so impaired the adhesive abilities of circulating cancer cells thereby increases their metastatic potential. We actually found that zoledronate significantly reduced mRNA expressions of α_4_-, α_6_- and β_1_-integrins in U2OS cells. In agreement with the previous results in the study, blockage of geranylgeranylation but not farnesylation reversed the zoledronate-inhibited adhesion to the collagen type 1 (α_2_β_1_-ligand) and gelatin (heat-denatured collagen, α_5_β_3_-ligand) in U2OS cells.

The more potent nitrogen-containing bisphosphonates (such as alendronate, ibandronate, and zoledronate) induce apoptosis in osteoclasts by inhibiting enzymes of the mevalonate pathway, including GGPP and FPP synthase [24]. The essential mevalonate pathway intermediate isoprenoids (GGPP and FPP) are essential for the post-translational farnesylation and geranylgeranylation of small GTPase, such as Rap, Rho and Ras [24]. Without isoprenylation, specific proteins are unable to attach into the membrane and do not function properly [35]. The Rho proteins (Rho, Rac, and Cdc42) are identified as tightly regulated molecular switches that cycle between an inactive, GDP-bound form and an active, GTP-bound form [12, 16, 36]. As previously reported [26, 34], and as in the study of our findings, zoledronate inhibits U2OS cell migration by decreasing Rho A and Cdc42 content and GTPγS activities in the cell membrane and increasing cytosolic Rho A and Cdc42 content.

Free nonsterol mevalonate pathway intermediates, FOH and especially GGOH, are capable of salvaging protein isoprenylation and, thus overcome statin and apoptosis induced by nitrogen-containing bisphosphonates in many cell types [24, 37-40]. For the correct function of these proteins, prenylation is required because the lipid prenyl group allows the anchorage of the proteins in cell membranes and may also participate in protein-protein interactions [41]. Accordingly, depletion of the formation of essential mevalonate pathway intermediate isoprenoids and the loss of prenylation of small GTPases (preferentially geranylgeranylated) by nitrogen-containing bisphosphonates could account for the various effects observed on cancer cells like our findings in the study through specific blockage of geranylgeranylation by zoledronate in U2OS cells [40]. As evidenced by the study, geranylgeranylation but not farnesylation, of these small GTPases is required for their binding to the cellular membrane in order to be active [42].

Many prenylated proteins are small G-proteins that are integral components of various signal transduction pathways in cancer cells. For example, farnesylation of oncogenic Ras is essential for cancer transformation [43]. Similarly, activation of the Rho family of small G-proteins such as Rho A and Rac1 require geranylgeranylation for their biological function, coupled with activation of the Raf/MAPK pathway, leads to oncogenic Ras transformation [44]. The overwhelming evidence implicating the importance of activation of the small G-proteins prompted us to further identify them using the synthesized GGTase I and FTase inhibitors. GGTI-298 and FTI-277 are C*AAX* peptidomimetics that potently and selectively inhibit and GGTase I and FTase to block geranylgeranylation and farnesylation respectively [45]. Intriguingly, GGTI-298 mimicked the effects of zoledronate, whereas FTI-277 had no effects. This further indicated that zoledronate blocks geranylgeranylation of small GTP-binding proteins via inhibition of the GPP synthase not farnesylation via inhibition of the FPP synthase in the mevalonate pathway, which leads to a decline of Rho A and Cdc42 prenylation and FAK-inhibited JNK and p38 pathways, to suppress U2OS cells invasion and migration. The anti-metastatic effects of zoledronate on U2OS cells are inhibited by geranylgeranylation rather than farnesylation, as previously mentioned by others [40].

Integrins are believed to involve in various intracellular pathways, including those involved in cell adhesion, migration, polarity, survival, growth, and death, suggesting their importance in cancer [46]. Expression of the α_6_β_4_-integrin complex promotes tumor progression and metastasis of various cancer cells, including breast, colorectal, and thyroid carcinomas [47]. Increased expression of α_5_-, β_1_- and β_3_-integrins correlating with poor survival of patients with early non–small cell lung cancer has been reported [48]. Importantly, β_1_-integrin signaling has been shown to activate diversely in cancer progression including invasion, migration and metastasis [12]. Although anti-adhesive effects of zoledronate have been described in breast cancer and human umbilical vein endothelial cells [26, 49], the underlying molecular mechanisms in osteosarcoma cells have not been studied in detail. We investigated the effects of zoledronate on α_4_-, α_6_- and β_1_-integrins reduction in U2OS cells and the inhibition of cell adhesion to the collagen type 1 (α_2_β_1_-ligand) and gelatin (heat-denatured collagen, α_5_β_3_-ligand) in the context of mevalonate pathway suppressed by inhibited geranylgeranylation, but not farnesylation. In combination with the previously described effects on motility, migration and invasion by zoledronate, the anti-adhesive abilities may add the influence on the distant metastasis of U2OS cells negatively. Further validation of the role of these genes and pathways is needed.

This study has several limitations. First, the physiological relevance of the experimental concentrations of zoledronate used in the *in vitro* study may not be calculated accurately *in vivo*. Second, it is difficult to detect the absorption, transportation and distribution of zoledronate in various tissues of the body, especially the levels of zoledronate in the osteosarcoma tissues clinically. As prescribing information from Novartis Pharmaceuticals Corporation, East Hanover, NJ 07936, after intravenous injection, approximately 22% zoledronate bound in human plasma, independent of the concentration, but zoledronate does not inhibit human P450 enzymes *in vitro* and does not undergo biotransformation *in vivo*. Third, zoledronate may inhibit different pathways in various malignant tumors and 4 osteosarcoma cell lines [11, 32, 33]. However, zoledronate is used widely for the treatment of patients with hypercalcemia of malignancy, patients with multiple myeloma, and patients with documented bone metastases from solid tumors. It is achievable for 50 μM zoledronate clinically. Even more than the concentration range exhibiting cytotoxicity, anti-tumor effects may develop and provide a great benefit to the patient with osteosarcoma despite anti-metastasis property [50].

Herein, we first clarify the importance of cell morphology change, EMT and cytoskeletal rearrangement in processes regulating cellular motility, migration and invasion, and provide insight into the mechanism of zoledronate-suppressed Rho A activation effect in U2OS cells to inhibit JNK and p38 pathways. We identify the temporal relationship of zoledronate-mediated Rho A and Cdc42 content between translocation from cytosol to membrane and small GTPase loading and the relative importance of Rho and Cdc42 in regulating U2OS cellular motility, migration and invasion. We further investigate the zoledronate-suppressed adhesive activity on U2OS cells by impairing α_4_-, α_6_- and β_1_-integrins expressions, which may contribute the negative influence on distant metastasis. Finally, we determine the anti-metastatic effect of zoledronate on U2OS cells by blocking geranylgeranylation, but not farnesylation. Certainly, our work reinforces the idea that zoledronate possesses the anti-metastatic properties on osteosarcoma cells, contributing to a better understanding of the mechanism responsible for these effects.

## MATERIALS AND METHODS

### Cell culture and zoledronate treatment

The Saos2 (osteogenic sarcoma) cells (11-yr-old Caucasian female), which were obtained from American Type Culture Collection (Manassas, VA, USA), were cultured in Dulbecco's Modified Eagle Medium (DMEM; Invitrogen Corp., Life Technologies, Carlsbad, California, USA) supplemented with 10% fetal bovine serum (FBS; Hyclone Laboratories, Inc-Logan, UT) and 1% penicillin (100 U/mL)/streptomycin (100 μg/mL) (Sigma, St. Louis, MO, USA). MG-63 cells (human, 14-yr-old male), which were obtained from the Food Industry Research and Development Institute (Hsinchu, Taiwan), were cultured in minimum essential medium (Gibco BRL, Grand Island, NY, USA) supplemented with 10% FBS, 1 mM glutamine, 1% penicillin/streptomycin, 1.5 g/L sodium bicarbonate, 0.1 mM non-essential amino acids, and 1 mM sodium pyruvate (Sigma, St. Louis, MO, USA). HOS (human osteosarcoma; 13-yr-old female) cells, which were obtained from the Food Industry Research and Development Institute (Hsinchu, Taiwan), were cultured in Eagle's MEM medium supplemented with 10% FBS, 1% penicillin/streptomycin, and 5 mL glutamine. U2OS (osteogenic sarcoma; human, 15-yr-old female) cells, which were obtained from the Food Industry Research and Development Institute (Hsinchu, Taiwan), were cultured in DMEM supplemented with 10% FBS, 1% penicillin/streptomycin, and 5 mL glutamine. The cell culture was maintained at 37°C in a humidified atmosphere of 5% CO_2_ incubator. Zoledronate [2-(imidazol-l-yl)-1-hydroxyethylidene-1,1-bisphosphonate] was provided by Novartis Pharma (Basel, Switzerland).

### Microculture tetrazolium (MTT) assay

For the cell viability experiment, a MTT (3-(4,5-dimethylthiazol-2-yl)-2,5-diphenyltetrazolium bromide) colorimetric assay was performed to determine the cytotoxicity of different concentrations (0, 25, 50, 75, and 100 μM) of zoledronate. After the exposure period, the media were removed and the cells were washed with phosphate-buffered saline (PBS). Afterwards, the medium was changed and the cells were incubated with MTT (0.5 mg/mL) for 4 h [5].

### Wound healing assay

To study the possibility that zoledronate alters motility of 4 osteosarcoma cells, we plated 9 × 10^5^ Saos2 cells, 8 × 10^5^ MG-63 cells, 1 × 10^6^ HOS cells and 9 × 10^5^ U2OS cells in 6-well plates for 16 h, wounded by scratching with a pipette tip, then incubated with DMEM containing 0.5% FBS and treated with different concentrations (0, 25, 50, 75 and 100 μM) of zoledronate for 0, 6, 12, and 24 h. Cells were photographed using a phase-contrast microscope (×100) as described elsewhere [51, 52]. To examine whether the farnesylated and geranylgeranylated proteins are involved in the zoledronate-induced inhibition *in vitro* wound closure, the mevalonate or the mevalonate-derived isoprenoids were added, including GGOH and FOH.

### Cell invasion and migration assays

To test the effect of zoledronate on the invasiveness of 4 osteosarcoma cells *in vitro*, we used a modified Boyden chamber invasion assay with Matrigel coating [5, 51, 52]. After treatment with the indicated concentrations of zoledronate (0, 25, 50, 75, and 100 μM), 4 osteosarcoma cells were seeded into the upper section of the Boyden chamber (Neuro Probe, Cabin John, MD, USA) at a density of 4 × 10^4^ cells per well, and then incubated for 24 h at 37°C. Finally, they were counted under a light microscope. Migration of cells treated with indicated concentrations of zoledronate (0, 25, 50, 75, and 100 μM) was measured as described in the cell invasion assay without Matrigel coating [5, 51, 52]. To examine whether the farnesylated and geranylgeranylated proteins are involved in the zoledronate induced inhibition in cell invasion and migration assays, GGOH and FOH were added.

### Gelatin zymography

To explore whether zoledronate suppresses MMP-2 and MMP-9 activities in 4 osteosarcoma cells, gelatin zymography was used. After plating 8 × 10^4^ Saos2 cells, 8 × 10^4^ MG-63 cells, 8 × 10^4^ HOS cells, and 8 × 10^4^ U2OS cells in 24-well plates for 16 h, cells were treated with different concentrations (0, 25, 50, 75, and 100 μM) of zoledronate for 24 h. Precast sodium dodecyl sulfate–polyacrylamide gels containing 0.1% gelatin were prepared and electrophoresis was done, and then gels were processed as described elsewhere [5, 51, 52].

### Immunocytofluorescence

To determine the effect of zoledronate on cell morphology, EMT and cytoskeletal organization, we utilized the antibody-based immunofluorescence staining method. U2OS cells (8 × 10^4^ cells per well) were cultured on glass coverslips and grown for 16 h so that they attached to the surface of the coverslips completely and 50 μM zoledronate was added. Cells were grown at 37°C in humidified 5% CO_2_ for 24 h. After washing with PBS 3 times, cells were fixed with 4% paraformaldehyde in PBS solution for 20 min and permeabilized with 0.1% Triton X-100 in PBS for 10 min. After washing with PBS, cells were blocked with 5% bovine serum albumin in PBS for 1 h. Then the samples were incubated with Texas Red-X phalloidin (200 U/ml) in 1% bovine serum albumin in order to localize filamentous actin (F-actin) for 1 h. For E-cadherin and FAK, the samples were incubated with mouse-anti-E-cadherin (1:250) and mouse-anti-FAK (1:500) at 4°C overnight. Subsequently, the samples were washed with PBS and incubated with FITC-conjugated mouse antibody for 1 h at 37°C. The nuclei were counterstained with 4′-6-diamidino-2-phenylindole (DAPI) for 5 min. The coverslips were then washed extensively and mounted on glass slides with mounting medium (DAKO, Glostrup, Denmark) for quantitative image analysis. The image of samples coverslips were examined with Zeiss LSM 510 META confocal microscope. To examine whether the farnesylated and geranylgeranylated proteins are involved in the cell morphology and cytoskeletal organization disturbed by the zoledronate, GGOH and FOH were added.

### Preparation of cell lysates and membrane proteins and western blot analysis

To investigate the molecular mechanism further, signaling pathways were detected using western blot analysis. After treatment with different concentrations of zoledronate (0, 10, 20, 30, 40, and 50 μM) for 24 h, the total cell lysates of 2.1 × 10^6^ U2OS cells were prepared as described elsewhere [5, 51, 52]. For extracting membrane proteins from the cell lysate Mem-PER eukaryotic membrane protein extraction reagent kit was purchased from Thermo Scientific, IL, USA. Following the manufacturer's instructions, 3 × 10^6^ U2OS cells after 24 h treatment of 50 μM zoledronate were prepared to harvest. Finally, the majority of membrane protein would be in the lower hydrophobic fraction, which could be used for membrane protein analysis. Conversely, the residue upper hydrophilic fraction could be used for analysis of cytosolic proteins. Samples including cell lysates, membrane proteins and cytosolic protein extraction were incubated with the primary antibodies, washed and monitored by immunoblotting using specific secondary antibodies. To examine whether the farnesylated and geranylgeranylated proteins are involved in the protein expression disturbed by the zoledronate, GGOH and FOH were added.

### Reverse transcriptase–polymerase chain reaction (RT-PCR)

Total RNA was extracted from U2OS cells using a guanidinium chloride procedure. Complementary DNA (cDNA) synthesis and PCR amplification were performed. Specific primers were used for Slug, Snail and Twist genes. For Slug, Snail, Twist and glyceraldehyde-3-phosphate dehydrogenase (GAPDH), the following forward (F) primers, and reverse (R) primers were used: Slug-F: gAgCATTTgCAgACAggTCA, R: CCTCATgTTTgTgCAggAgA; Snail-F: TTCTCCCgAATgTCCCT, R: TCAgCCTTTgTCCTgTAgC; Twist-F: AgTCCgCAgTCTTACgAggA, R: CATCTTggAgTCCAgCTCgT; GAPDH-F: CggAgTCAACggATTggTCgTAT, R: AgCCTTCTCCATggTTggTgAAgAC. To examine whether the farnesylated and geranylgeranylated proteins are involved in the m-RNA level disturbed by the zoledronate, GGOH and FOH were added, including GGOH and FOH.

### Interfering RNA

For silencing E-cadherin, Rho A and Cdc42 proteins expression, small interfering RNA (siRNA) inhibiting human E-cadherin (s2770), Rho A (s759), Cdc42 (s55424) and negative-control siRNA (4390844) were purchased from Applied Biosystems Instruments (Foster City, CA, USA). 2.1 × 10^6^ U2OS cells were grown on 6 cm cell culture dishes overnight. According to the manufacturer's instructions, a total of 30 pM of E-cadherin siRNA, Rho A siRNA and Cdc42 siRNA were transfected into the cells using lipofectamine RNAiMAX reagent (Invitrogen, Carlsbad, CA, USA). The Silencer negative control siRNA, a nonsense siRNA duplex, was used as a control.

### Rho A and Cdc42 activation assay

To identify whether Rho A and Cdc42 are active and bounding to GTP or inactive and bounding to GDP, we purchased active Rho and Cdc42 pull-down and detection kits, respectively (Thermo Scientific, IL, USA). Based on the manufacturer's instructions, we used 500μg to 1 mg of total cell lysate to specifically pull down active Rho A and Cdc42 for western blot detection of Rho A and Cdc42 activation. Two control nucleotides, GTPγS and GDP, could be used to generate positive and negative control lysates, respectively.

### Microarray analysis

2.1 × 10^6^ U2OS cells were treated with zoledronate at indicated concentrations (0, 50, 100 μM) for 6 hours. The cells were harvested by TRIzol reagent and total RNA were analyzed according to the manufacturer's instructions of Human OneArray from Phalanx Biotech (Hsinchu, Taiwan). The results were analyzed by the Rosetta Resolver System. Compared with the control sample, the fold change of integrin family gene expression is established at log2 ratio and enrichment analysis was established at log10 ratio and *p* < 0.05, using gene ontology analysis.

### Adhesion assay

Collagen, gelatin and fibronectin (0.1 μg/ml, 100 μL/well) were coated on 24-well plates at 37°C overnight and then the wells were washed and blocked in 3% bovine serum albumin. After treatment with or without 50 μM of zoledronate, U2OS cells were detached from culture by trypsin. Cells (1 ml; 1 × 10^5^ cells) were added to the wells and incubated at 37°C overnight. The adherent cells were stained with 0.1% Crystal Violet in 25% methanol for 30 minutes. Wells were then washed and dried and s then olubilization of the Crystal Violet was performed with 0.1 M sodium citrate containing 50% ethanol. Absorbance was measured at 590 nm (microplate reader, Bio-Tek Instruments, Inc., Winooski, VT, USA). To examine whether the farnesylated and geranylgeranylated proteins are involved in the m-RNA level disturbed by the zoledronate, GGOH and FOH were added, including GGOH and FOH.

### Statistical analysis

For all of the measurements, analysis of variance followed by one-way ANOVA with post hoc Bonferroni test was used for more than two groups. Each experiment was performed in triplicate and 3 independent experiments were performed. *p* values < 0.05 was considered statistically significant.

## SUPPLEMENTARY FIGURE


