# Supplementary material for: Zoledronate blocks geranylgeranylation not farnesylation to suppress human osteosarcoma U2OS cells metastasis by EMT via Rho A activation and FAK-inhibited JNK and p38 pathways
Source: Oncotarget. 2016 Feb 2;7(9):9742–58. doi: 10.18632/oncotarget.7138 (PMC4891081; doi:10.18632/oncotarget.7138)
Supplement: Supplementary file 1 [file oncotarget-07-09742-s001.pdf]

SUPPLEMENTARY FIGURE

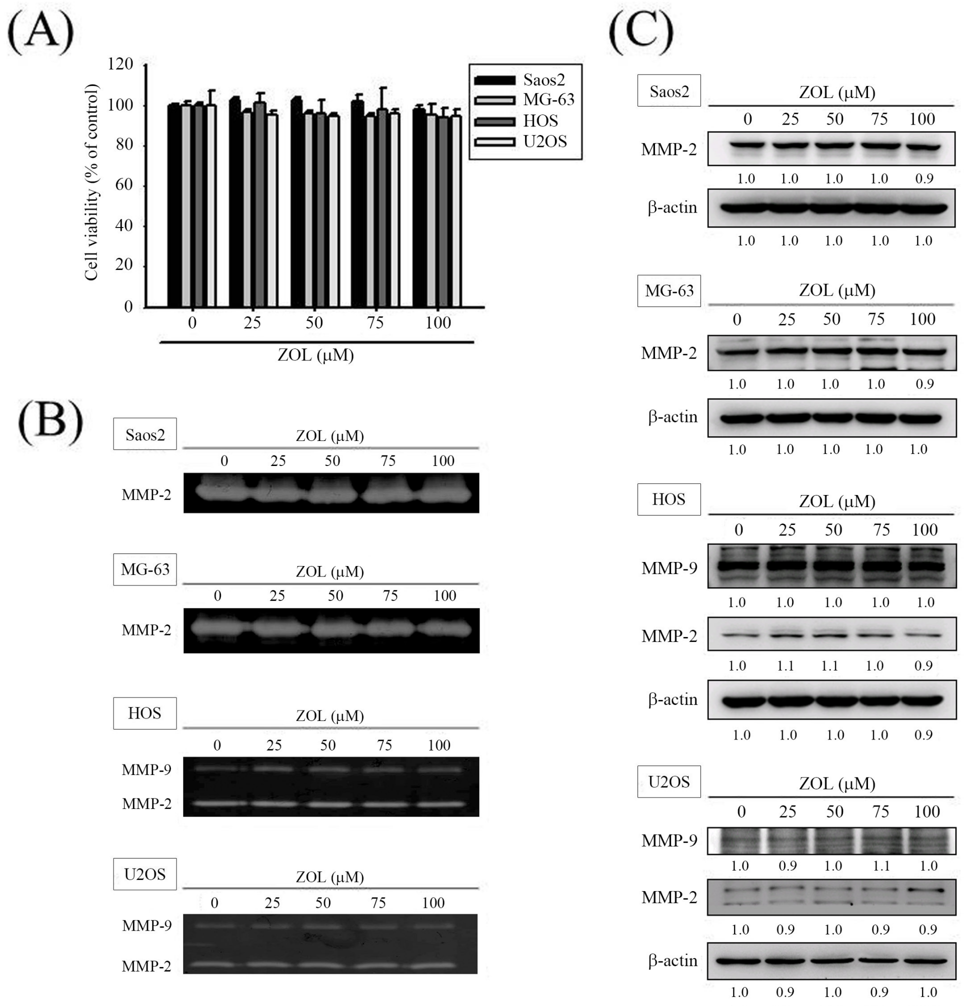

Supplementary Figure S1: Effects of zoledronate on cell viability and gelatinases in 4 osteosarcoma (Saos2, MG-63, HOS and U2OS) cells. A. Four osteosarcoma cells were treated with zoledronate (0, 25, 50, 75 and 100 µM) for 24 h before being subjected to a MTT assay for cell viability. Analysis of variance was used. Saos2: $F = 3.086$, $p = 0.068$; MG-63: $F = 2.536$, $p = 0.084$; HOS: $F = 1$, $p = 0.486$; U2OS: $F = 0.392$, $p = 0.81$. **B.** Activities and **C.** protein expressions of MMP-2 and MMP-9 after different concentrations (0, 25, 50, 75, and 100 µM) of zoledronate treatment in 4 osteosarcoma cells were measured by gelatin zymography and western blot analysis, respectively.
